# Clinical predictors of significant intracranial computed tomography scan findings in adults experiencing headache disorder

**DOI:** 10.11604/pamj.2020.35.81.16041

**Published:** 2020-03-19

**Authors:** Ulrich Igor Mbessoh Kengne, Callixte Kuate Tegueu, Dorothée Soh Mankong, Maggy Mbede, Ulrich Gael Tene, Boniface Moifo

**Affiliations:** 1 Faculty of Medicine and Biomedical Sciences, The University of Yaounde 1, Yaounde, Cameroon; 2 Neurology Department, Douala Laquintinie Hospital, Douala, Cameroon; 3 Radiology Department, Yaounde Central Hospital, Yaounde, Cameroon; 4 Radiology Department, Yaounde Gynaeco-Obstetric and Pediatric Hospital, Yaounde, Cameroon

**Keywords:** Headache disorder, clinical predictors, head computed tomography scan, intracranial findings

## Abstract

**Introduction:**

Radiological assessments for adult headache disorder show significant intracranial findings in 2.5% to 10% of performed computed tomography scans (CT-scans), leading to an overuse consideration for CT-scan requests by physicians in headache-experiencing patients. Therefore, we undertook this study in order to determine predictors of significant intracranial CT-scan findings in adults experiencing headache disorder; in order to help physicians better select patients who need imaging, which would subsequently decrease the costs of headache disorder management and the useless irradiation rates.

**Methods:**

We carried out a cross-sectional study in the medical imaging departments of Yaounde Central Hospital and Douala Laquintinie Hospital, which are two teaching hospitals in Cameroon, over a period of five months. We consecutively and non-exhaustively included all consenting patients aged eighteen years and above, referred to the radiology department to perform a head CT-scan as aetiological workup of headache disorder, from either a traumatic or non-traumatic mechanism. Patients having a known brain lesion and those with a Glasgow coma scale less than thirteen were excluded. The clinical history of patients was taken and a complete physical examination was performed. Demographic data, clinical characteristics of the headache, results of neurological and physical examinations were collected and correlated to the results of head CT-scan.

**Results:**

We enrolled 169 patients in the study, 56.2% were males, with a sex ratio of 1.3; sudden onset of headache increased by two the risk of discovering significant intracranial pathology (p = 0.032). Occipital and cervical location of headache, headache evolving by crisis, and recurrent paroxysmal headache were rather significantly correlated to no structural brain finding. An abnormal neurological examination with specifically abnormal stretch reflexes, aphasia, loss of consciousness, raised intracranial signs, weakness, and meningeal signs were predictive of structural intracranial pathology. Otorrhagia, epistaxis, and periorbital ecchymosis in addition were predictive in post-traumatic headaches.

**Conclusion:**

Abnormal results from neurological examination are the best clinical parameters to predict structural intracranial pathology on CT-scan in adult patients experiencing headache disorder. In case of post-traumatic headaches, in addition, otorrhagia, epistaxis, and periorbital ecchymosis are too highly predictive.

## Introduction

Headache or cephalalgia is defined as localized or diffused pain in various parts of the head, with the pain not confined to the area of distribution of a nerve, and eventually irradiating to the face or the neck [1,2]. Headache disorder is among the most common pain problem encountered in daily practice[1,3]. With a life-time prevalence estimated at 99% for women and 94% for men [4], it is designated in many literature as the most frequent neurological symptom leading adult people to consult a physician in emergencies [2,5,6]. The World Health Organisation (WHO) ranges headache disorder among the ten major world-wide pathological conditions [7-9]. Thus, headache disorder today is a global public health problem, due to numerous disabilities and financial implications of the management. International Headache Society (HIS) classifies headache into primary (without any organic substratum) or secondary (with organic substratum) according to many criteria in order to select which patients suffering from headache disorder will need CT-scan or magnetic resonance imaging (MRI) imaging workup [1,10]. Brain imaging for adult suffering from headache disorder show significant intracranial findings in 2.5% to 10% of CT-scans [5,11]. Yet, CT-scan has the greatest radiation exposure in imaging [12]; and impertinent requests will lead to unnecessary radiations with their consequences [13], and a financial burden for the patients [14,15]. Magnetic resonance imaging (MRI), which is the gold standard in the evaluation of headache disorder [6], is available only in big cities like Yaounde or Douala with very high cost in our setting. In sub-Saharan Africa, and specifically in Cameroon, data on clinical factors predicting abnormal CT-scan findings in headache disorder, are scarce. Therefore, we undertook a study on predictors of significant intracranial CT-scan findings in adults suffering from headache disorder; in order to help physicians to better select patients with secondary headache who need imaging, and subsequently reduce the cost of headache care.

## Methods

**Setting and period:** we carried out a cross-sectional study in medical imaging departments of Yaounde Central Hospital and Douala Laquintinie Hospital, which are two teaching hospitals in Cameroon, from January to May 2017.

**Inclusion criteria:** we included all consenting patients aged eighteen years or above, referred to the Radiology Department to perform a head CT-scan as aetiological workup of headache disorder, from either traumatic or non-traumatic mechanism.

**Exclusion criteria:** patients having a known brain lesion and those with a Glasgow coma scale less than thirteen were excluded.

**Data collection:** after informed consent of eligible patients, we took their clinical history and performed a neurological examination and a complete physical examination before they undergo a brain CT-scan. The CT-scan was performed in helical mode with or without contrast enhancement according to the clinical context, beginning from C2 vertebra to the whole cranium, and with a millimetric and multiplan reconstruction. The interpretation was performed in both cerebral and bone windows by experimented radiologists, and the examination of the facial block and cervical occipital hinge was systematically done. The CT-scan results were classified in significant intracranial findings, which could be a cause of headaches; incidental findings and normal results were considered as non-significant findings. From each consenting patient, socio-demographic as well as past medical history, full general and neurological examination information were collected, clinical characteristics of headache and significant intracranial findings. Additionally, we studied the predictive values of clinical signs to get a significant intracranial finding in adult patients suffering from headache disorder.

**Statistical analysis:** data analysis used the Statistical Package for Social Science (SPSS) Version 23. Group's comparison used the chi square tests and variants, after stratification of continuous variables as appropriate. Continuous variables were reported as mean (SD) when the distribution was normal and as median (interquartile range) when the distribution was not normal. Categorical variables were analyzed using χ^2^ and Fisher exact tests as appropriate. Logistic regression models were used to investigate the predictors of significant CT-scan findings, with adjustment for potential confounders. The significance level of all tests was p ≤ 0.05.

**Ethical approval:** this study got approval from the Research and Ethics Institutional Committee of the Faculty of Medicine and Biomedical Sciences.

## Results

One hundred and sixty nine patients were enrolled in the study; 56.2% were men, the sex ratio was 1.3. The mean age was 43.05±16.7 years. One hundred and thirty two patients (78.1%) performed head CT-scan for acute headaches, of which 45.6% were recent sudden-onset headache and 32.5% were recent progressive headache. The general characteristics of the study group are shown in Table 1. With regard to the relationship between the characteristics of headache and the findings on CT-scan, headache of sudden-onset were statistically associated with the risk of abnormal findings on CT-scan (p = 0.032) On the other hand, recurrent headache, those of occipital and cervical location, and recurrent paroxysmal headache were statistically associated with the absence of significant CT-scan finding (Table 2). An abnormal neurological examination in patients suffering from headache disorder (p = 0.018); aphasia (p = 0.006); periorbital ecchymosis after head trauma (p = 0.023) and abnormal reflexes (p = 0.026) were statistically associated with abnormal CT-scan findings (Table 2). Loss of consciousness (69.6%), raised intracranial pressure signs (70%), sensitivomotor weakness (72.7%), meningeal irritation signs (81.8%), and post-traumatic otorragia (100%), were positive predictive clinical signs of having abnormal intracranial findings on brain CT-scan of adult patients suffering from headache disorder (Table 3).

**Table 1:** Distribution of socio-demographic data and headache’s clinical presentation in the sample

Characteristics		N (%)
		(Years)
**Age**	Mean age	43.05±16.7
	Median	40
	Range	18-91
**Gender**	Male	95 (56.2)
	Female	74 (43.8)
**Characteristics of headache**	Recent sudden-onset headache	77 (45.6)
	Recent progressive headache	55 (32.5)
	Recurrent paroxysmal headache	29 (17.2)
	Chronic daily headache	8 (4.7)

**Table 2:** Correlation between clinical signs and significant CT-scan findings

Clinical signs		Significant CT-scan findings		Total N(%)	OR	P-value
		Yes n (%)	No n (%)			
Characteristics of headaches	Sudden onset	57 (62.6)	34 (37.4)	91 (53.8)	1.96 (1.06-3.62)	0.032
	Evolution in crisis	36 (45.0)	44 (55.0)	80 (47.3)	0.46 (0.25-0.85)	0.013
	Occipital and cervical location	8 (28.6)	20 (71.4)	28 (16.6)	0.26 (0.11-0.64)	0.002
	Recurrent paroxysmal headache	11 (37.9)	18 (62.1)	29 (17.2)	0.43 (0.19-0.98)	0.042
Clinical signs associated to headaches	Abnormal neurological examination	73 (60.8)	47 (39.2)	120 (71)	2.25 (1.14-4.43)	0.018
	Aphasia	21 (91.3)	2 (8.7)	23 (13.6)	7.11 (1.51-33.6)	0.006
	Abnormal stretch reflexes	26 (83.9)	5 (16.1)	31 (18.3)	3.47 (1.12-10.7)	0.026
	Peri orbital ecchymosis (in case of head trauma)	9 (90.0)	1 (10.0)	10 (5.9)	8.13 (1.01-65.71)	0.023
	Epistaxis (in case of head trauma)	10 (83.3)	2 (16.7)	12 (7.1)	5 (1.03-24.11)	0.030

**Table 3:** Predictive values of clinical signs on detecting significant brain CT-scan findings in adults suffering from headache disorder

Clinical signs	Significant brain CT-scan findings	
	Positive predictive value (%)	Negative predictive value (%)
Post-traumatic otorrhagia	100.0	18.2
Meningeal signs	81.8	30.3
Sensitivomotor weakness	72.7	38.5
Signs of raised intracranial pressure	70.0	46.4
Loss of consciousness	69.6	31.4
Recent sudden-onset headache	61.0	50.0
Recent progressive headache	58.2	46.5
Seizure	43.8	37.6
Chronic daily headache	33.3	43.8

## Discussion

In this study involving patients referred to the Radiology Department to perform a head CT-scan as aetiological workup of headache disorder in two tertiary care hospital in sub-Saharan Africa where data are sparse, we report new data on clinical predictors of abnormal CT-scan findings. All patients included in the study for headache disorders underwent a brain CT-scan after full physical end neurological examination. Headache of sudden onset as well as patients with abnormal physical examination like aphasia, abnormal tendon reflexes, those with periorbital ecchymosis or epistaxis after head trauma were statistically associated with abnormal findings on brain CT-scan. A variability is observed in many studies about the sociodemographic profile of the sample included, closely due to the inclusion or not of headache disorder consecutive to a traumatic mechanism. Our sample was made of 56.2% of males, similarly to 56% of males in the study of Ndabahweje *et al.* [16]. Indeed, in many towns of low income countries of sub-Saharan Africa, the road traffic abounds of motor drivers with motor bikes as the main mode of transportation, and those motor bikes are driven mainly by men, therefore exposing them to brain trauma. In other hand, some professional activities are also at risk of causing brain trauma. Additionally, the epidemiological studies revealed that brain trauma are mainly attributed to younger adults [17-19]; almost half of the patients in our sample were aged less than 40 years. In studies with few or any case of post-traumatic headache disorders like those of Sonhaye *et al.* and Kouame *et al*. a female predominance was observed [5,20]. Recent sudden-onset headache and recent progressive headache were the headache's clinical presentation which mostly induced head CT-scan requests in our study. These were described respectively by 45.6% and 32.5% of patients. The study of Sonhaye *et al.* showed similar observations [5]. According to the literature, acute headache presentation alone or associated with other clinical signs denominated as “red flags” are warning signs that might lead physicians to suspect a serious intracranial pathology like tumors, hemorrhagic stroke and therefore the request for brain CT-scan or MRI [1,21-23].

We report in this study that headache of sudden onset significantly multiplies by two the risk of having an abnormal finding on brain CT-scan. On the other hand, acute headache disorders are still a source of worry for the patients who subsequently ask their physician to order neuroimaging in order to alleviate their anxiety and for physicians too, due to the fear of missing a serious raised intracranial lesion [1,10,24]. Headaches evolving by crisis, of occipital and cervical location and recurrent paroxysmal headaches characteristically denominate clinical semiology of primary headache disorders, such as migraine or tension-type headaches [25]. Yet, as primary headache disorders don't have any organic substratum visible on neuroimaging [1,10], we found that they significantly increased the risk of normal brain CT-scan. Valenca *et al.* have also demonstrated that 61.5% of CT-scan requested for migraine or tension-type headaches were of no value [25]. Adult patients suffering from headache disorder experience other clinical signs of which we have identified those which are predictive of brain CT-scan abnormalities. An abnormal neurological examination significantly increases the risk of showing a lesion on brain CT-scan and a positive correlation was found with aphasia and abnormal myotatic reflexes. Other neurological positive predictive signs were loss of consciousness, signs of meningeal irritation, sign of raised intracranial pressure, and any neurological weakness. Likewise, studies from Mitchell *et al.* and Kajal *et al.* found that all patients of whom brain CT-scan revealed significant intracranial findings had an abnormal neurological examination [26,27]. In patients complaining of headache disorder after a minor head trauma, periorbital ecchymosis and epistaxis significantly increased the risk of abnormal finding on brain CT-scan and otorrhagia where highly predictive. Those results are corroborative with literature, who showed that the occurrence of nasal or auricular bleeding, loss of consciousness and weakness after a brain trauma would lead to a head CT-scan request. So, the occurrence of those clinical signs in patients experiencing headache disorders will increase the value of head CT-scan requests.

## Conclusion

Adult patients experiencing headache disorder must be scrupulously and carefully questioned and examined to bring out the headache's characteristics and other associated clinical signs that may be warnings of an intracranial morbid process. The consideration of imaging for headache-experiencing patients with abnormal neurological examination, nasal or auricular bleeding, and periorbital ecchymosis after a minor head trauma, will substantially improve the value of head CT-scan in the management of headaches in daily practice.

### What is known about this topic

- Abnormal neurological exam is an indication of neuroimaging explorations in adult patients experiencing headache disorder;
- CT-scan assessments for headache disorders in adult patients mostly lead to either normal results or no significant findings.

### What this study adds

- Positive and negative predictive values of relevant clinical signs to find a significant pathology on CT-scan in adult patients experiencing headache disorder;
- Clinical characteristics of headaches correlated to significant CT-scan findings;
- Clinical characteristics of headaches correlated to normal results or no significant CT-scan findings.

## Competing interests

The authors declare no competing interests.
